# A Parsimonious Granger Causality Formulation for Capturing Arbitrarily Long Multivariate Associations

**DOI:** 10.3390/e21070629

**Published:** 2019-06-26

**Authors:** Andrea Duggento, Gaetano Valenza, Luca Passamonti, Salvatore Nigro, Maria Giovanna Bianco, Maria Guerrisi, Riccardo Barbieri, Nicola Toschi

**Affiliations:** 1 Department of Biomedicine and Prevention, University of Rome Tor Vergata, 00133 Rome, Italy; 2 Department of Information Engineering and Research Centre “E. Piaggio”, University of Pisa, 56122 Pisa, Italy; 3 Institute of Bioimaging and Molecular Physiology, National Research Council, 20090 Milano, Italy; 4 Department of Clinical Neurosciences, University of Cambridge, Cambridge CB2 0QQ, UK; 5 Department of Experimental and Clinical Medicine, Magna Graecia University, 88100 Catanzaro, Italy; 6 Department of Health Sciences, Magna Graecia University, 88100 Catanzaro, Italy; 7 Department of Electronics, Informatics and Bioengineering, Politecnico di Milano, 20133 Milano, Italy; 8 Department of Radiology, Martinos Center for Biomedical Imaging and Harvard Medical School, Boston, MA 02129, USA

**Keywords:** Granger causality, directed brain connectivity, MEG connectivity, laguerre polynomials

## Abstract

High-frequency neuroelectric signals like electroencephalography (EEG) or magnetoencephalography (MEG) provide a unique opportunity to infer causal relationships between local activity of brain areas. While causal inference is commonly performed through classical Granger causality (GC) based on multivariate autoregressive models, this method may encounter important limitations (e.g., data paucity) in the case of high dimensional data from densely connected systems like the brain. Additionally, physiological signals often present long-range dependencies which commonly require high autoregressive model orders/number of parameters. We present a generalization of autoregressive models for GC estimation based on Wiener–Volterra decompositions with Laguerre polynomials as basis functions. In this basis, the introduction of only one additional global parameter allows to capture arbitrary long dependencies without increasing model order, hence retaining model simplicity, linearity and ease of parameters estimation. We validate our method in synthetic data generated from families of complex, densely connected networks and demonstrate superior performance as compared to classical GC. Additionally, we apply our framework to studying the directed human brain connectome through MEG data from 89 subjects drawn from the Human Connectome Project (HCP) database, showing that it is able to reproduce current knowledge as well as to uncover previously unknown directed influences between cortical and limbic brain regions.

## 1. Introduction

Granger causality is a widely applied model-based tool to quantify directed information transfer between signals originating from (possibly) complex networks in a number of disciplines, ranging from econometrics to neuroscience. In particular, the study of the so-called brain “connectome” (i.e., the entirety of all multivariate functional interactions between brain areas that can be possibly estimated starting from neuromonitoring time-series data such as Magnetoencephalography (MEG), electroencephalography (EEG) or functional magnetic resonance imaging (fMRI)) has provided fertile ground for the application of most diverse causality-based methods [1,2,3,4,5,6]. Often, connectomics studies in general, and causality estimation in neuroscience in particular, are planned in a network-discovery fashion, i.e., no strong a priori hypotheses exist about which causal links or sub-networks should be expected and/or studied. However, typical neuromonitoring data are often characterized by data paucity as compared to the number of channels necessary for whole-brain coverage. While fMRI represents an extreme case of this problem (with hundreds of thousands of voxels, i.e., signal sources, for a few thousand data points), neuroelectric signals like EEG or MEG (which are commonly recorded with much higher frequencies as compared to fMRI) are not immune from the same issue. Indeed, signal nonstationarity and the high density of MEG and EEG artifacts often mandates estimation within short signal epochs. At the same time the physiological relevance of long-range correlations in brain signals is becoming more and more evident [7,8,9,10,11].

In the context of large multivariate systems, the classical multivariate autoregressive Granger causality-based formulation (MVAR-GC) may therefore suffer from important limitations due to the approximate quadratic dependence of the number of parameters on the system’s dimensionality, especially in relation to the number of available datapoints. In these cases, approaches like e.g., “partial conditioning” [12,13,14], may be more relevant/useful than full conditioning. Additionally, methods dealing with data redundancy, possibly determined by an influence that is external to the system, have been devised [15]. Another important aspect is the possible presence of long range correlations (see above) and/or putative non-instantaneous, possibly delayed influences between regional brain signals. Here, approaches able to estimate or take into account characteristic interaction at different time-scales have been proposed, based on e.g., state-space [16,17] or on wavelet formulations [18]. When such long-range correlations are present, the optimal autoregressive order in MVAR estimation (and hence the number of model parameters) will have to grow further in order to be able to capture enough information from the system’s past to faithfully represent signal dynamics.

A number of analytical and algorithmic methods have been targeted specifically to the reduction of model parameters. Such approaches are generally based on strategies to optimally select a subset of possible alternative models, and employ more or less sophisticated search strategies such as stepwise methods [19,20], genetic algorithms [21], particle swarm optimization [22,23], or least absolute shrinkage and selection operators (LASSO) [24,25,26]. Still, the complexity of these methods decreases generality, calls for application-specific optimization and generally comes with high computational demands.

Other approaches have tackled the problem from a data reduction perspective, i.e., rather than shrinking model parameter space, signal space is compressed/embedded into a component subspace which is assumed to represent most of the non-redundant information contained in the original signals [2,27]. While this may lead to efficient model estimation, when applying such approaches the estimated causal relationships will be related to this reduced network of (latent) components rather than to the original signals, possibly hampering interpretability.

In view of the widespread application of Granger causality to biosignals and neuroscience in general, in the rest of this paper we will refer to “causality” in the classical Granger-sense. Still, it is important to note that the definition of causality provided by Granger is still controversial, and that there is a lively scientific debate about (possibly) alternative definitions and ideas about causal inference based on more recent and/or sophisticated frameworks. As an example, the ideas introduced though Pearl’s interventionist do-calculus [28] (a leading concept of contemporary philosophy in which causality is represented through the concepts of action-intervention-manipulation) recently facilitated the development of modern approaches to estimating causality based on e.g., symbolic dynamics and algorithmic probability [29]. These applications are particularly relevant when e.g., developing a machine-oriented tool [30] based on reasonable functional assumptions stemming from structural equation models (like e.g., in econometrics or molecular biology—please see Discussion for additional details).

In this study, we aimed to overcome the above issues while remaining within the realm of linear models, for which parameter estimation remains a convex problem. To this end, we introduce the use of a Wiener–Volterra decomposition with Laguerre polynomials as basis functions. This choice allows to build parsimonious MVAR models [31,32,33,34,35] while capturing arbitrarily long past dependencies without increasing the number of parameters. Specifically, the orthonormal basis of the discrete-time Laguerre functions expand the Wiener–Volterra kernels, accounting for the long-term information in correlated time series with a possible heterogeneous delay structure in their interdependence [31,33,35]. We then reformulate classic causality estimators in terms of this decomposition, validate our method in synthetic data and report an example application MEG data drawn from the large Human Connectome Project (HCP) database.

## 2. Theory

**Figure 1:**
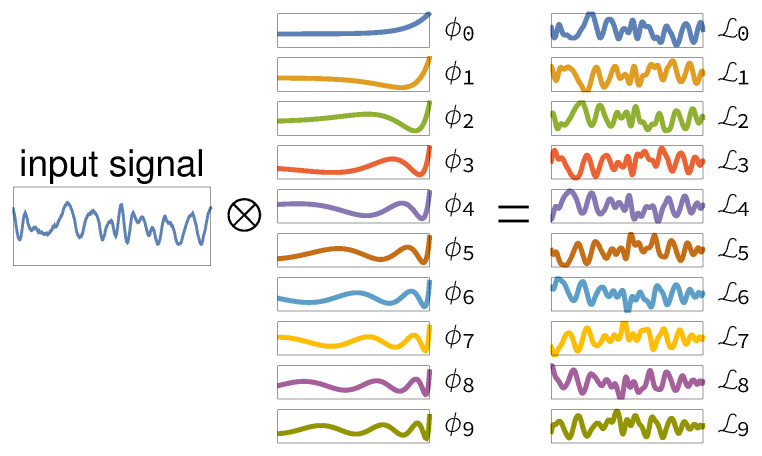
Example of the use of Laguerre basis functions in compactly representing signals: the input signal $x_{i}$ is convolved in time with functions $\phi_{m}$ to obtain the function $L_{i}^{m}$, which is then used in autoregressive modeling.

When formulating the problem of detecting causality from variable $Y_{t}$ to variable $X_{t}$ (Y→X) one tests the null hypothesis that the knowledge about the past of $Y_{t}$ provides no added prediction power when forecasting the future of $X_{t}$. We reformulate the classical “restricted” model (RM) and “unrestricted” model (UM) which are at the bases of causality estimation using a Laguerre expansion. We call this approach Laguerre-based Granger causality (LGC) (an earlier version has been presented in [36]). The RM for $X_{t}$, includes the past of $X_{t}$ itself and $Z_{t}$. The latter term includes all other variables except for $Y_{t}$ (i.e., the model is “conditioned”). Further, the UM includes all variables $X_{t}$, $Y_{t}$, $Z_{t}$ [4]. Both models are fitted using MVAR systems which, in this paper, are defined over the components of a Volterra–Wiener expansion with Laguerre polynomials:

(1) $$\begin{array}{ccc} x_{t+1} & =\sum_{k=1}^{p}a_{k}L_{t}^{\left(k\right)}\left(x\right)+c_{k}L_{t}^{\left(k\right)}\left(z\right)\;+\varepsilon_{t}\;\; & \left(\mathrm{RM}\right) \end{array}$$

(2) $$\begin{array}{ccc} x_{t+1} & =\sum_{k=1}^{p}{\tilde{a}}_{k}L_{t}^{\left(k\right)}\left(x\right)+{\tilde{b}}_{k}L_{t}^{\left(k\right)}\left(y\right)+{\tilde{c}}_{k}L_{t}^{\left(k\right)}\left(z\right)\;+\tilde{\varepsilon_{t}} & \left(\mathrm{UM}\right) \end{array}$$

(see Figure 1) where *p* is the autoregressive model order, *a*, $\tilde{a}$, $\tilde{b}$, *c*, $\tilde{c}$ are autoregressive coefficients, ε, $\tilde{\varepsilon }$ are uncorrelated white noise processes, and the discrete-time Volterra–Wiener decomposition with Laguerre polynomials $L^{\left(m\right)}\left(\cdot \right)$ over the discrete time signal $x_{t}$ is defined as:

(3) $$L_{t}^{\left(m\right)}\left(x\right)=\sum_{n=0}^{N}\phi_{m}\left(n\right)\left(x_{t-n}\right)$$

where the mth-order, discrete time Laguerre polynomial $\phi_{m}\left(n\right)$ is defined as [34,37]:

(4) ϕm(n)=αn−m2(1−α)12∑j=0m(−1)jnjmjαm−j(1−α)j.

Here, the parameter α(0≤α<1) determines the rate of exponential asymptotic decay of $\phi_{m}\left(n\right)$ [38] (see Figure 1 for a visual example).

Notably, the null-hypothesis that $Y_{t}$ does not cause $X_{t}$ (conditioned to $Z_{t}$), can then be expressed as the an equality between the expectation of $x_{t}$

(5) $$E\left(x_{t}|Z_{t-k}\right)=E\left(x_{t}|Y_{t-k},Z_{t-k}\right),$$

and therefore rejected if the $f_{\mathrm{ratio}}$ of the residual sum of squares (RSS):

(6) $$f_{\mathrm{ratio}}=\frac{RSS_{r}-RSS_{\mathrm{ur}}}{RSS_{\mathrm{ur}}}\frac{N_{\mathrm{obs}}-2m}{m},$$

is extreme with respect of its parent distribution, i.e., the Fisher–Snedecor distribution with $N_{\mathrm{obs}}-2m$ and *m* degrees of freedom [3]. In this context, it is common practice to employ the logarithm of the ratio of average squared residuals as a measure of GC strength as follows [39]:

(7) $$s_{j\to i}=\log\left(\frac{{RSS}_{r}}{{RSS}_{\mathrm{un}}}\right)\;\;.$$

Also, the *p*-value related to the above hypotheses test can be expressed analytically if needed [40]. Still, the use of Equation (7) has several advantages: (i) in the limit of large strengths it is proportional to −log(p−value), which, in turns is normality distributed with a mean proportional to the number of independent observations [41]; and (ii) it can be easily be generalized to the case of multivariate GC [4,39].

## 3. Methods

In this paper we explore the performance of the above described LGC, and compare it to the classical MVAR-GC employing massive multivariate synthetic data simulation from random networks. In-house developed code employed for LGC is available at: https://github.com/andreaduggento/Laguerre-GrangerCausality.

### 3.1. Network Generation

**Figure 2:**
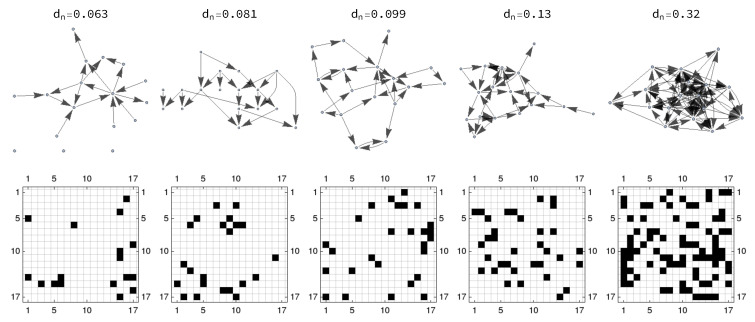
Example networks (for a subset of density values) used to generate synthetic data. (**top row**) Topological directed representation; (**bottom row**) corresponding adjacency matrices (white fields are zeros, black fields are ones).

In order to compare the performances of LGC and MVAR-GC in detecting true causal connections within complex directed networks, we performed synthetic data simulation by generating data from families of 17-node, ground-truth networks described by a binary, zero-diagonal, asymmetrical adjacency matrix A, whose elements $A_{ij}$ represent the direct influence of node *j* on node *i*. Pairs of nodes with bidirectional connections as well as loops are explicitly allowed. The total number of “edges” $n_{e}$ depends on the desired network density $d_{n}$ which, for a network with *L* nodes, is defined as $n_{e}\left(L\left(L-1\right)\right)$. We generated graph families at 19 different densities, where the values of $d_{n}$ are chosen to be approximately equidistant on a logarithmic scale between 0.01 and 0.5. For each value of $d_{n}$, we generated 32 different random networks to account for possible fluctuations of our estimator with respect to network topology. Examples of the generated networks and their corresponding adjacency matrices for exemplary density values are shown in Figure 2.

### 3.2. Synthetic Simulations

Each node in the network families described above was assigned a system which is coupled to other nodes through the adjacency matrix described above (see below for details), and evolved as a stochastic variable characterized by long range correlations. The coupling terms were characterized through integral relationships which include random delays (see below). Each stochastic variable $x_{i}$ is a sum of three components: (i) a noise term which exhibits long-range correlation behaviour $\xi_{i}$; (ii) a cubic integral term; (iii) a coupling term which feeds from other nodes:

(8) $$x_{i}\left(t\right)=d\xi_{i}\left(t\right)-h\left(\int_{t-h}^{t}\frac{x_{i}^{3}\left(s\right)}{3}ds+c_{ij}\left(\eta_{j}\left(t-\tau_{j}\right)-x_{i}\left(t\right)\right)\right),$$

where $\tau_{j}$ is a delay term which is randomly sampled at each realization from a uniform distribution in the range 0–12 s, d=0.1, and the time step is set to h=0.25 s. Each coupling coefficient $c_{ij}$ is defined by a global, overall coupling strength w=0.2 as well as by the the ground-truth adjacency matrix A. Specifically, for the *i*-th node, if for all *j*$A_{ij}=0$, then $c_{ij}=0$; otherwise $c_{ij}$ is equal to *w* normalized by the number of incoming connections $c_{ij}=w/\sum_{j}A_{ij}$.

The noise term is the main determinant of the the statistical properties of $x_{i}$ and it is designed to include long-range correlation properties [42]

(9) $$\begin{array}{cc} \xi_{i}\left(t\right) & =\sum_{l=1}^{w}-a_{l}\xi_{i}\left(t-l\right)+d\epsilon_{i}\left(t\right) \end{array}$$

(10) $$\begin{array}{cc} a_{l} & =\left(l-1-\frac{\beta }{2}\right)\frac{a_{l-1}}{l}, \end{array}$$

where *w* is the noise-model size (set to w=3 in this paper), $a_{0}$ = 1, ϵ(t)∼N(0,1) is a normally distributed random variable, and β characterizes the noise spectrum. The latter follows a power law $1/f^{\beta }$ and does not have a characteristic timescale [43]. Also, the cubic integral term $x^{3}$ guarantees that the variable $x_{i}$ remains symmetrically bounded around 0. Finally, the coupling term $c_{ij}\left(\eta_{j}\left(t-\tau_{j}\right)-x_{i}\left(t\right)\right)$ is defined by the coupling coefficient $c_{ij}$ and an integral term delayed by $\tau_{j}$ for of the *j*-th variable:

(11) $$\begin{array}{cc} \frac{d}{dt}\eta_{j}\left(t\right) & =-\eta_{j}/\tau_{0}+bx_{j}. \end{array}$$

**Figure 3:**
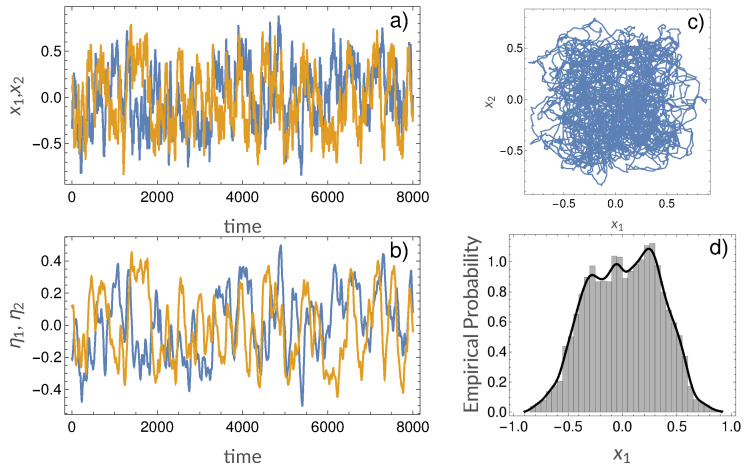
Example realizations of our model system: (**a**) example signals $x_{i}$; (**b**) example signals $\eta_{i}$; (**c**) phase plot of $x_{1}$ vs. $x_{2}$; (**d**) typical distribution of $x_{i}$.

In our simulations we set the time decay constant $\tau_{0}=8$ and b=0.1. Each network was evolved for a total of 10,000 time-points, and the generated multivariate time series were employed for caulsality analysis, using both the LGC and MVAR-GC approaches. For the MVAR-based GC method, the autoregressive model order was chosen according to the Akaike information criterion [44] resulting in an optimal order p=10. The autoregressive order was employed to model all variables of the system. An example of signal generated from Equations (8)–(11) is presented in Figure 3. For each set of multivariate signals (i.e., for each network realization) and corresponding estimated adjacency matrix, the ability of LGC and MVAR-GC to detect causal links (i.e., belonging to the ground truth network) while rejecting false causal links was quantified as the area under the receiver operating characteristic (ROC) curve (AUC). The AUC was computed by varying the threshold in causality strength $s_{j\to i}$ in Equation (7) which determined, for every pair of nodes *i* and *j*, whether their causal connection should be accepted as “true” or rejected. Additionally, estimation was performed while varying parameter α within the interval α∈[0,0.7] in order to investigate the relationship between α and AUC.

### 3.3. Directed Brain Connectivity Estimation in MEG Data

As an example application of our method to biological signlas, we use resting-state magnetoencephalography (rMEG) data from 89 subjects made available by the Human Connectome Project (HCP) [45] as part of the S1200 release (https://www.humanconnectome.org/study/hcp-young-adult/document/1200-subjects-data-release).

Every subject included in this dataset underwent 3 sessions of 6-minutes rMEG scans, along with physiological data were also acquired, including electrooculography (EOG), electrocardiography (ECG), and electromyography (EMG). These data were employed by the HCP consortium in denoising and preprocessing the data. In brief, the MEG preprocessing pipeline begins with several steps of data sanity check and quality assurance tasks (see https://www.humanconnectome.org/storage/app/media/documentation/s1200/HCP_S1200_Release_Reference_Manual.pdf for details).

Data were purged of artifacts resulting in excessive signal amplitude, and “bad” channels were selected through the estimation of the correlation between each channel and its neighbors [46]. Raw MEG data was then separated into brain-related and noise components through an independent component analysis (ICA) pipeline which included an automatic classification step using a manually trained expert classifer. The ICA pipeline included (i) band-pass filters; (ii) ECG and EOG preprocessing; (iii) iterative ICA decompositions (20 iterations of FastICA with deflation approach run from different initializations); (iv) power spectrum density and power time course estimation; (v) parameter estimations to allow classification of environmental and instrumental artifacts. Successively, source estimation was performed using a single shell volume conduction model defined in a MEG-system-based head coordinates obtained by segmentation of the anatomical magnetic resonance imaging (MRI) scan for each subject. Finally, time-varying estimates of the band-limited (8 frequency bands) power envelope of each source are generated (final signal frequency: 40 Hz). The frequency ranges for each band are summarized in Table 1.

Successively, the power envelopes were aggregated according to the 17 cortical parcels described in [47]. This resulted in 136 (17 parcels × eight bands) six-minute power envelopes per subjects. The physiological interpretation of the functional networks/parcels employed are summarized in Table 2. Given the notable skewness of the data, each power envelope was log-transformed prior to multivariate LGC estimation. The latter was performed subject- and band-wise, after which the third percentile in strength across all subject was extracted for visualization purposes.

## 4. Results

**Figure 4:**
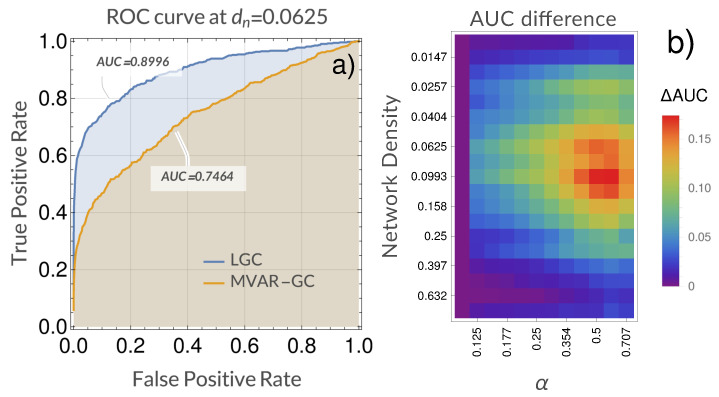
Comparison of detection performance between the classical multivariate autoregressive Granger causality (MVAR-GC) and Laguerre-based Granger causality (LGC) for our model system. (**a**) Example area under the receiver operating characteristic (ROC) curves (AUCs) resulting from using both classical MVAR-GC and LGC (α=0.595) for a single network density. ROC curves shown on the left were built over the prediction of $(17^{2}-17)\times 32=8704$ links relative to 32 random networks with density $d_{n}=0.0625$. (**b**) Difference between AUCs (defined as ΔAUC = AUC(LGC) − AUC(MVAR-GC) as a function of network density and α. As in the ROC curves on the left, every ΔAUC value (corresponding to every pair of density and α values) in the figure on the right is built over all links belonging to 32 random networks.

Figure 4 shows an example of the comparison between the ROC curves obtained when using LGC vs MVAR-GC for density $d_{n}=0.0625$ (approximately 0.9 vs 0.75 in AUC), as well as the gain in performance (i.e., AUC) obtained when using LGC as opposed to classical MVAR-GC as a function of network density and α. For this system, a qualitatively optimal (in the sense of performance gain over MVAR-GC) region of α centered around α=0.6 emerged almost across the whole density range.

**Figure 5:**
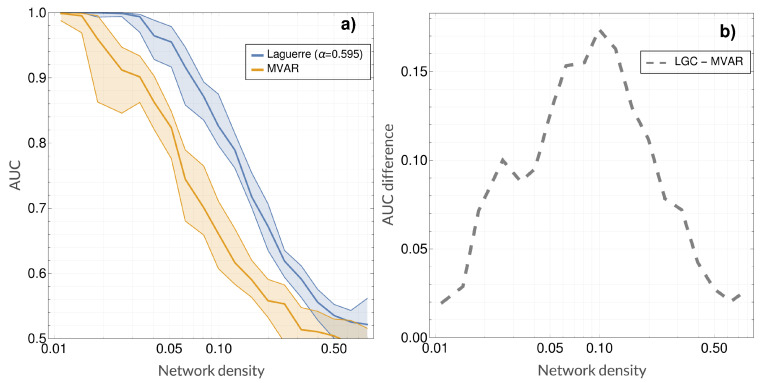
Comparison of detection performance between the classical multivariate autoregressive Granger causality (MVAR-GC) and Laguerre-based Granger causality (LGC) for our model system. (**a**) Median AUC values (along with interquartile ranges calculated across 32 random networks for each density) as a function of density for both LGC and MVAR-GC; (**b**) performance gain as the difference between median AUC values for LGC and MVAR-GC as a function of density. The value for α was chosen according to Figure 4.

**Figure 6:**
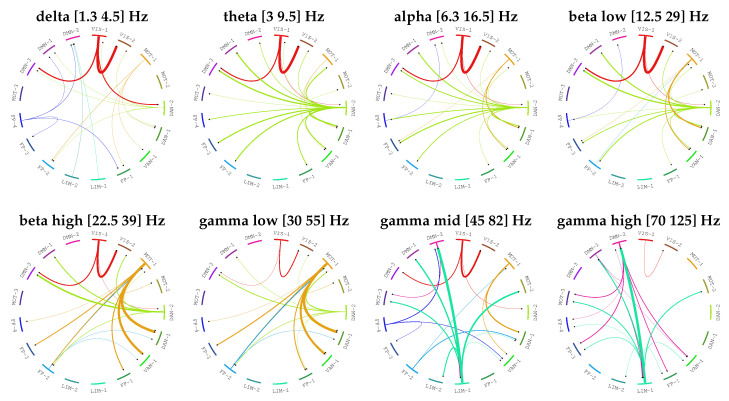
Results of employing LGC to quantify the directed, magnetoencephalography (MEG)-based connectome in the high quality Human Connectome Project (HCP) sample. For each band, only the top 3% connections (median strength across subjects) are shown. Every edge (i.e., connection) is colored according to the node (i.e., network) which is driving the other node. The width of each edge if proportional to the strength of the connection.

Additionally, Figure 5 shows AUC values (along with interquartile ranges) as a function of density for both LGC and MVAR-GC. Here, the value for α was chosen according to Figure 4. Performance gain is also shown on the right as a function of density. As expected, the discrimination performances degrade monotonically when network density (and therefore the complexity of the problem) increases. Both classical MVAR-GC and LGC reach AUC = 1 (100% accuracy) for very low network densities, and both methods approach AUC = 0.5 (chance-level discrimination accuracy) for very densely connected networks. However, for every density tested, LGC performed better than MVAR-GC, and its discrimination performance appears to degrade less steeply as network density increases. The maximum performance gain was approximately 0.17 (difference in AUC), which was attained at a network density of approximately 0.1 (which, incidentally, corresponded to a typical threshold values employed in connectomic studies). Finally, Figure 6 shows the results of employing LGC to quantify the directed, MEG-based connectome in the high quality HCP sample. For each frequency band, only the top 3% connections (median strength across subjects) are shown. The 3% threshold was arbitrary chosen in order to compromise between including physiologically-relevant information while minimizing the number of possibly false positive causal links.

## 5. Discussion and Conclusions

In this paper, we have proposed a method to compactly represent and model signals in a linear autoregressive framework through a novel orthogonal basis based on Laguerre polynomials. This novel formulation allows us to attain model parsimony while retaining the ability to represent long-range correlation, a fundamental requirement when modeling high-frequency brain signals. This approach was employed to revise classical, MVAR model based Granger causality and validated in large scale simulation using synthetic data generated from families of complex networks at varying densities. A clear advantage of the LGC methods was shown across all densities. This is in line with the idea that Laguerre polynomials are smooth basis functions, able to capture damped multiple-frequency oscillations with fewer parameters when compared to classical MVAR models. In order to mimic the complexity of biological signals, our synthetic simulations were based on a nonlinear system with integral couplings as well as random delays, however without the presumption that it could serve as a thorough synthetic generative model for MEG data. It is thus possible to hypothesize that one of the several nonlinear causality estimation methods presented in the literature [48,49,50,51,52] could deliver even better performance. However, such methods require articulate parameter optimization, and their identification may not be univocal. It should be noted that the advantages of LGC with respect of MVAR-GC may only apply to certain situations, e.g., when significant delays or slow couplings at multiple timescales are present. However, as mentioned above, this is very often the case with physiological signals derived from the brain as well as from other physiological subsystems. Also, interestingly, the MVAR system defined through Laguerre base functions can be simply viewed as a generalization of the classical MVAR system; indeed, the latter is simply recovered by setting α=0. In this sense, this work could be viewed as proposing and extension to classical, linear multivariate causality by introducing the additional choice of the optimal α (with 0≤α<1), which only introduces one additional degree of freedom with a large gain in signal representation ability. In this sense, introducing an α > 0 could be viewed as an attractive alternative to incrementing the model autoregressive order *p*.

It is important to note that the Granger-style approach might not be the most suitable tool to accurately investigate the complex causal interplay between the phenomena underlying observed dynamics, and that recent literature points towards a revised definition fo the term “causal” which does not necessarily include Granger approaches. For example, recently the ϵ-transducer has been exploited to provide a structural representation of the minimal optimal predictor of one process from another [53,54]. Also, based on the recent theoretical formulation of partial information decomposition (PID) by Williams and Beer [55] the scientific community is currently debating what the most appropriate definitions of redundant, unique and synergistic components of mutual information should be (for a review, see [56,57] and references therein). While redundancy measures a form of equivalence [58], alternative definitions have emerged both for shared information [59,60,61] and for unique information [62]. It is interesting to note that several recent tool which have been presented in order to tackle the ideas of redundancy and synergy in information within time series rely on autoregressive representations, and may therefore benefit from the representational basis we have presented in this paper in order to minimize parameter count at implementation time.

Following synthetic data validation, we applied LGC to estimate the directed connectome of the human brain through a specific 17-dimensional parcellation of a high-quality MEG dataset provided by the HCP consortium. This was intended as a data-driven, whole brain exploration of the directed, MEG-based connectome of the human brain. In view of the peculiar characteristics of the MEG signal (presence of long-range correlations), this application is well matched to the circumstances for which our method was designed. Also, while this approach, like any time-series analysis tool applied to biological systems, can be susceptible to higher order biological as well as data-driven confounders, it serves as a discovery tool which can help formulate more specific hypotheses (e.g., to be tested in targeted, tasks-based paradigms). Additionally, it is important to note that (a) each data channel employed in this paper contained approximately 14,000 data points, ensuring the feasibility of full conditioning, and (b) given the dangers of incurring in false positives when performing whole-brain, data driven explorations, only the top 3% connections resulting from our full multivariate analysis are presented and interpreted. Although the neurobiological basis and neuroanatomical localization of the different MEG bands remains a matter of debate, we found that our results are highly consistent with previous findings [63] showing that the theta band localizes to dorsal prefrontal networks, while the alpha and beta band respectively relate to neuronal activity in posterior occipital regions and motor cortices. The origin of gamma bands is controversial but has been linked to prefrontal networks and cognitive control [63].

More importantly, our data clearly show that the key brain regions previously implicated in specific neuronal rhythms are the same areas that causally drive the connectivity in other circuits (“nodes”) in each distinct band. For example, the primary occipital network (VIS-1) dominates the casually-driven connections in the alpha band, although an effect of the dorsal attentional network is also evident for the alpha and theta rhythms. Likewise, the motor circuit (MOT-1) directs the MEG-derived functional connectivity in ventral and dorsal attentional networks in the beta frequency domain. Increased beta activity over the motor cortex have been associated to voluntary movement suppression and can be dependent on the experimental procedures adopted in the study (i.e., resting-state MEG).

Finally, it is of particular interest to note the preponderant role of a limbic network (LIM-1) in mediating the activity in other circuits (especially the default mode network) at the gamma bands, particularly the highest frequencies. Previous studies have localized the origin of gamma bands to prefrontal regions and have suggested that gamma rhythms would reflect a synchronized firing of several circuits that would be critical to associate internal inputs (e.g., memories) with external (e.g., visual) ones to form coherent perception. Our findings support this idea by showing that the directed activity from limbic circuits to areas within the default mode network might play an important role in such high-level cognitive associative functions.

In conclusion, we have extended classical MVAR-GC to incorporate long-range past information and coupling while retaining model simplicity and linearity. In an application to real-world brain data, we have shown how our method is able to reproduce current knowledge as well as to uncover novel directed influences between brain regions. While the latter observations warrant additional validation through specific task-based investigation, in general we have shown that the LGC method is able to detect in vivo functional interactions and causal dynamics across multiple neural networks while delivering superior performance as compared to classical, linear MVAR-based Granger causality methods.

## Figures and Tables

**Table 1:** Frequencies band resulting from magnetoencephalography (MEG) preprocessing pipeline executed by the Human Connectome Project (HCP) consortium.

Frequency Band Name	Frequency Band Ranges
delta	[1.3, 4.5] Hz
theta	[3, 9.5] Hz
alpha	[6.3, 16.5] Hz
beta low	[12.5, 29] Hz
beta high	[22.5, 39] Hz
gamma low	[30, 55] Hz
gamma mid	[45, 82] Hz
gamma high	[70, 125] Hz

**Table 2:** Legend of the 17 functional network from Yeo resting state network map along with their physiological interpretation.

	Network Name	Physiological Interpretation
1	VIS-1	Visual
2	VIS-2	
3	MOT-1	Motor
4	MOT-2	
5	DAN-2	Dorsal Attention
6	DAN-1	
7	VAN-1	Ventral Attention
8	FP-1	Frontoparietal
9	LIM-1	Limbic
10	LIM-2	
11	FP-2	Frontoparietal
12	FP-3	
13	FP-4	
14	MOT-3	Motor
15	DMN-3	Default Mode Network
16	DMN-1	
17	DMN-2	

## Abbreviations

The following abbreviations are used in this manuscript:

AUC	Area under ROC curve
ECG	Electrocardiography
EEG	Electroencephalography
EMG	Electromyography
EOG	Electrooculography
fMRI	Functional magnetic resonance imaging
HCP	Human Connectome Project
ICA	Independent component analysis
LASSO	Least absolute shrinkage and selection operator
LGC	Laguerre Granger causality
MEG	Magnetoencephalography
MVAR-GC	MultiVARiate Granger causality
RM	Restricted model
rMEG	Resting-state magnetoencephalography
ROC	Receiver operating characteristic
RSS	Residual sum of squares
UM	Enrestricted model
